# Supplementary material for: Fiber reinforced GelMA hydrogel to induce the regeneration of corneal stroma
Source: Nat Commun. 2020 Mar 18;11:1435. doi: 10.1038/s41467-020-14887-9 (PMC7080797; doi:10.1038/s41467-020-14887-9)
Supplement: Supplementary file 1 — Supplementary Information [file 41467_2020_14887_MOESM1_ESM.docx]

Supplementary Materials for:

**Fiber reinforced GelMA hydrogel to induce the regeneration of corneal stroma**

**By Kong et al.**

The schematic diagram of the experimental process is shown in Supplementary Figure 1.


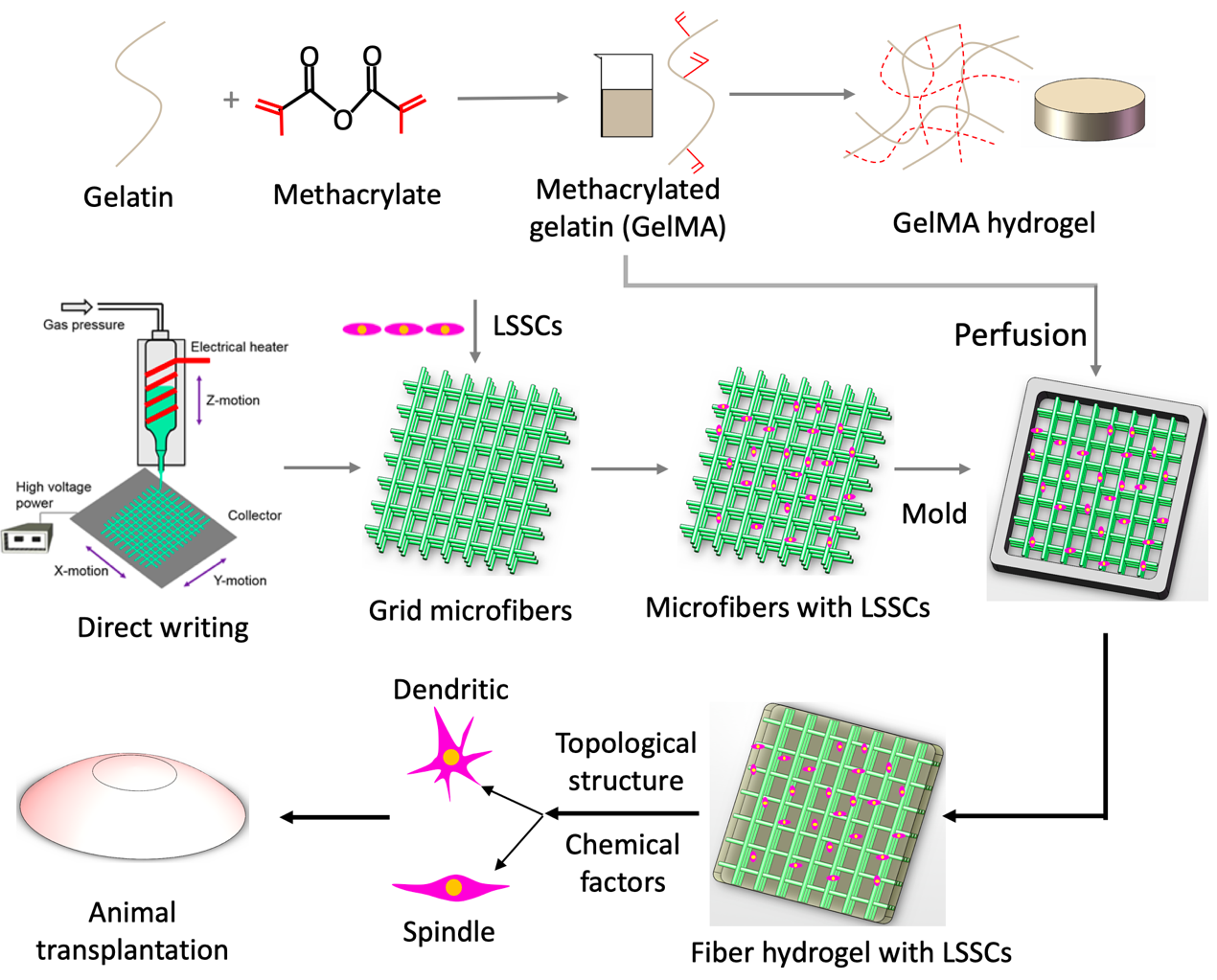


Supplementary Fig. 1 The schematic illustration of the experimental process.

PECL copolymers with a molecular weight (MW) of 8 WDa and 15 WDa were synthesized by the open ring polymerization of $\varepsilon$-CL monomers initiated by bio-functional HO-PEG-OH to increase the hydrophilicity of PCL. PCL with a molecular weight of 8 WDa initiated by cetyl alcohol was fabricated as a control (CPCL). The NMR and IR spectra were acquired to confirm the formation of copolymers, and the spectra of 8 WDa PECL copolymer with 10% and 1 WDa of PEG are shown in Supplementary Figure 2 and Supplementary Figure 3. From the NMR spectra, peaks at 1.648 (a), 2.309 (b), and 4.062 (d) were representing methylene protons in PCL unites, and the peak at 3.646 (c) was assigned to the methylene protons in PEG units. From the IR spectra, the absorption bands at 1720, 1170, and 1239 cm^-1^ were attributed to the C=O stretching vibrations of the ester carbonyl group, the C–O–C stretching vibrations of the repeated OCH_2_CH_2_ units of PEG, and the COO bonds stretching vibrations, respectively. These results indicated that the PECL copolymer was fabricated successfully. The MW of the PECL copolymer was calculated by the formula based on the integration ratio of CL methylene (d) and PEG methylene (c) indicated from the NMR spectrum.

DSC measurement was performed to determine the melting temperature (Tm) of the copolymer because the Tm is an essential parameter in the melting direct writing process. The DSC curves of PECL copolymer and CPCL shown in Supplementary Figure 4 indicated that the Tm of PECL was approximately 61$℃$, with is a slight decrease compared with the CPCL Tm of 63$℃$, which making it more favorable for direct writing.

The contact angle images and histogram of CPCL with MW of 8 WDa and PECL with MW of 8 WDa and 15 WDa are shown in Supplementary Figure 5; the addition of PEG can significantly reduce the contact angle of PCL, and the higher the PEG and PCL MW ratio, the lower the contact angle, indicating that the hydrophilicity of PCL was successfully improved by the synthesis of the PECL copolymer.

Swelling ability of hydrogels, which is influenced by hydrogel pore size, can indicate the degree of hydrophilicity and is an essential feature of the cornea. The measured mass swelling ration of the native cornea and pure GelMA hydrogel was 14.5±0.59 and 16.7±0.51, respectively. From the histogram shown in Supplementary Figure 6, the addition of grid fibers can reduce the swelling ratio of GelMA hydrogel, and the degree of reduction increases increased fiber spacing. This result may be because the micro-scale grid pore inhibits the increase of volume that was initiated by the permeation of water in the normal hydrogels. Compared with the remaining constructs, the swelling ratios of the 100G and 200G constructs were more similar to the native cornea at 13.9±0.49 and 14.6±0.25, respectively.

Sirius staining was used to determine the secretion of collagen fibers by the LSSCs after culturing in the 2 D culture dish and the 100 G fiber hydrogel under the SF media for 4 weeks, shown in Supplementary Figure 7. After staining, the collagen fibers would show red color and the nuclei of cells would show blue color under the normal microscope. From the results, we can find out that cells on both constructs secreted collagen fibers, which was consistent with the immunostaining results. Most collagen fibers secreted by cells in the 100 G construct distributed along the direction of the PECL fibers, but the collagen fibers secreted by cells in the culture dish showed random orientation, indicating that the aligned fibrous scaffold can induce the secretion of aligned extra cellular matrix of keratocyte.

Supplementary Fig. 2 Characterization of synthesized polymers: NMR spectrum of PECL copolymer.

Supplementary Fig. 3 Characterization of synthesized polymers: IR spectrum of PECL copolymer.


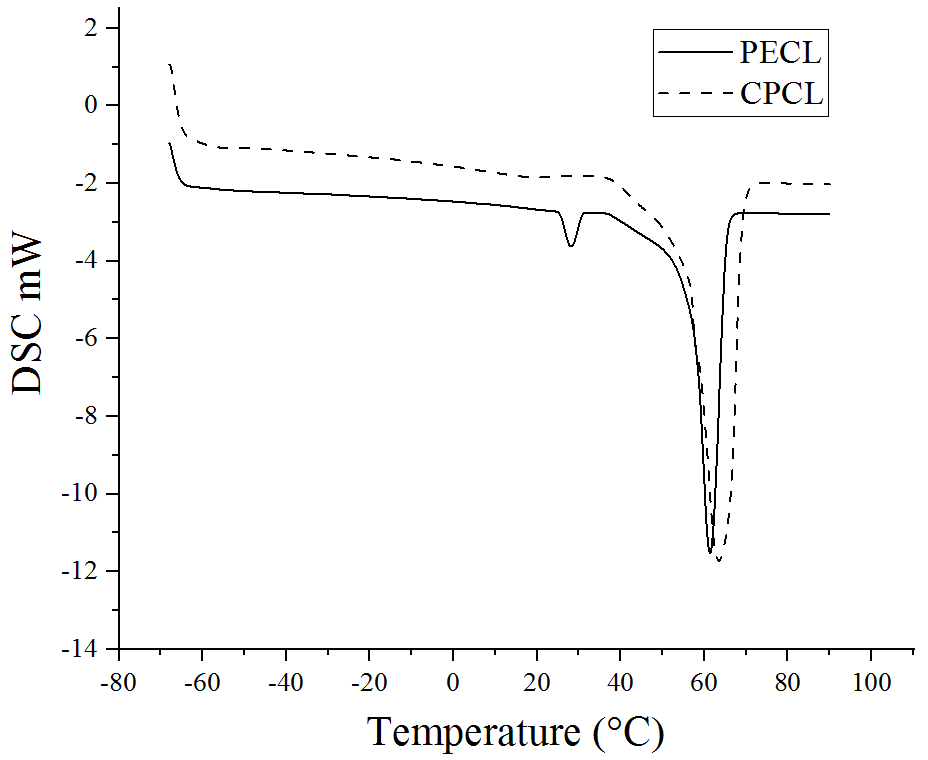


Supplementary Fig. 4 Characterization of synthesized polymers: DSC curves of CPCL and PECL

Supplementary Fig. 5 Characterization of synthesized polymers: Contact angle of CPCL and PECL with different molecular weights (n = 7, biologically independent samples). Data are presented as the average ± SD. ***P<0.001; ****P<0.0001; Unpaired two-tailed student’s t test.


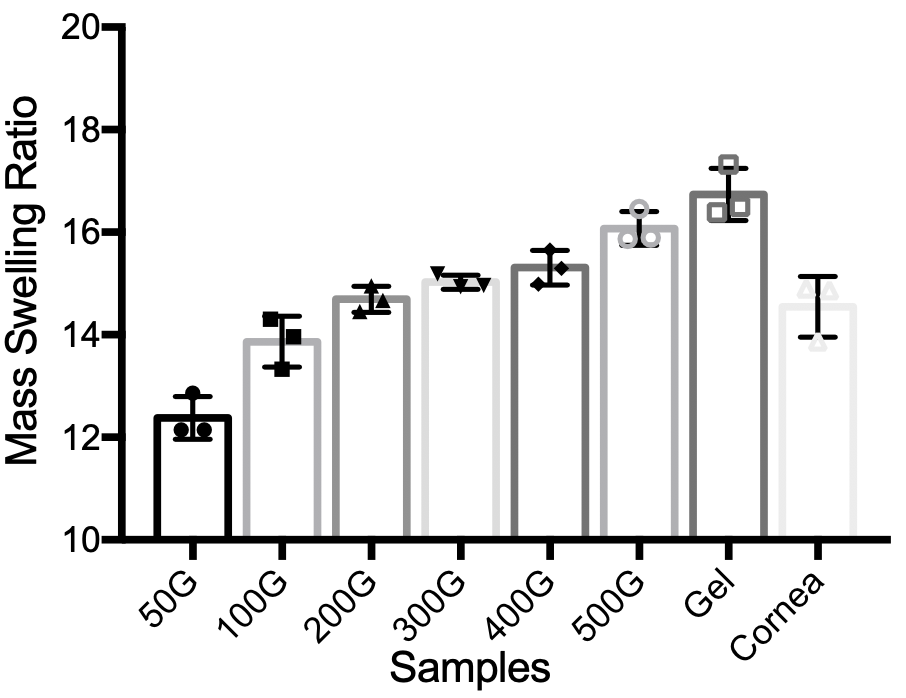


Supplementary Fig. 6 The swelling ratio histogram of 50G, 100G, 200G, 300G, 400G, and 500G constructs, the acellular cornea, and the pure GelMA hydrogel (n = 3, biologically independent samples). All the results were calculated as mean ± SD.


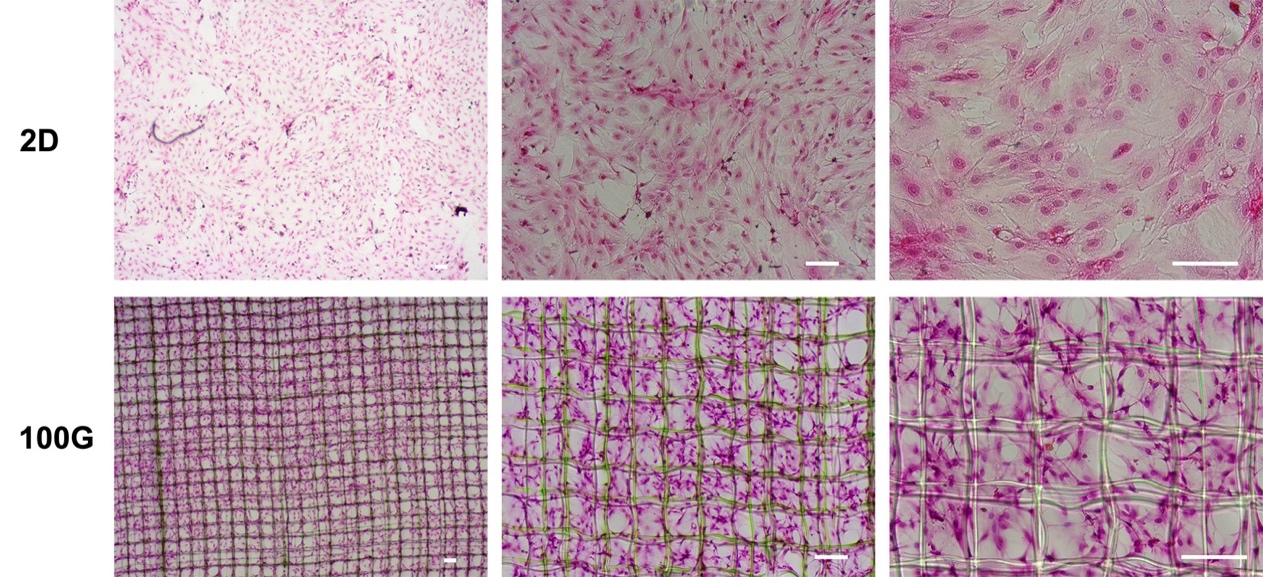


Supplementary Fig. 7 Sirius staining of collagen fibers secreted by LSSCs cultured in SF media on 2D TCPs and 100G constructs after culturing for 4 weeks. The scale bar is 100 um.

Supplementary Table 1 qPCR primer sequences.

| Gene | Sequence (5’-3’) |
| --- | --- |
| ALDH3A1  Forward  Reverse | CCAGTCCTCACGATACATAG  ATCACCTTCTCATTGTTGGA |
| KERATOCAN  Forward  Reverse | GACAACAACTCCATTGAAGG  CTGTAGGTTAGCATTGATTCG |
| AQP1  Forward  Reverse | ATCGTATGTGGCTCTTGGA  CGTTCGTGCGGTCTGTAA |
| THY1  Forward  Reverse | TACTCCACCACCAAGGATG  GCTTACGCCACCACACTTG |
| ACTIN  Forward  Reverse | AGATTACTGACTGGCTCCTA  CCCATACCCACCATCACACC |
