# Supplementary material for: Fiber reinforced GelMA hydrogel to induce the regeneration of corneal stroma
Source: Nat Commun. 2020 Mar 18;11:1435. doi: 10.1038/s41467-020-14887-9 (PMC7080797; doi:10.1038/s41467-020-14887-9)
Supplement: Supplementary file 2 — Reporting Summary [file 41467_2020_14887_MOESM2_ESM.pdf]

nature research

Corresponding author(s): Shengli Mi

Last updated by author(s): 2020/1/29

Reporting Summary

Nature Research wishes to improve the reproducibility of the work that we publish. This form provides structure for consistency and transparency in reporting. For further information on Nature Research policies, see Authors & Referees and the Editorial Policy Checklist .

Statistics

For all statistical analyses, confirm that the following items are present in the figure legend, table legend, main text, or Methods section.

n/a	Confirmed	
[ ]	[x]	The exact sample size (*n*) for each experimental group/condition, given as a discrete number and unit of measurement
[ ]	[x]	A statement on whether measurements were taken from distinct samples or whether the same sample was measured repeatedly
[ ]	[x]	The statistical test(s) used AND whether they are one- or two-sided<br>*Only common tests should be described solely by name; describe more complex techniques in the Methods section.*
[x]	[ ]	A description of all covariates tested
[ ]	[x]	A description of any assumptions or corrections, such as tests of normality and adjustment for multiple comparisons
[ ]	[x]	A full description of the statistical parameters including central tendency (e.g. means) or other basic estimates (e.g. regression coefficient) AND variation (e.g. standard deviation) or associated estimates of uncertainty (e.g. confidence intervals)
[ ]	[x]	For null hypothesis testing, the test statistic (e.g. *F*, *t*, *r*) with confidence intervals, effect sizes, degrees of freedom and *P* value noted<br>*Give P values as exact values whenever suitable.*
[x]	[ ]	For Bayesian analysis, information on the choice of priors and Markov chain Monte Carlo settings
[x]	[ ]	For hierarchical and complex designs, identification of the appropriate level for tests and full reporting of outcomes
[x]	[ ]	Estimates of effect sizes (e.g. Cohen's *d*, Pearson's *r*), indicating how they were calculated

Our web collection on statistics for biologists contains articles on many of the points above.

Software and code

Policy information about availability of computer code

Data collection	Data was collected using the following softwares:<br>-ToupView (Toup Tek Photonics)<br>- Xcellence (Olympus)<br>- Instron Bluehill (Instron)<br>- Phenomes-4.4 (Phenom)
Data analysis	Data was analyzed using the following softwares:<br>- Image Pro Plus 6.0<br>- Origin pro 2018c<br>- Matlab R2016a<br>- Prism 7 (GraphPad)

For manuscripts utilizing custom algorithms or software that are central to the research but not yet described in published literature, software must be made available to editors/reviewers. We strongly encourage code deposition in a community repository (e.g. GitHub). See the Nature Research guidelines for submitting code & software for further information.

Data

Policy information about availability of data

All manuscripts must include a data availability statement. This statement should provide the following information, where applicable:

- Accession codes, unique identifiers, or web links for publicly available datasets
- A list of figures that have associated raw data
- A description of any restrictions on data availability

The data that support the findings of this study are available from the corresponding author upon reasonable request.

Raw data of the following figures is attached in the supplementary source data: Figure 2B, Figure 2C, Figure 2D, Figure 2E, Figure 2F, Figure 3C, Figure 3D, Figure 3E, Figure 3F, Figure 3G, Figure 3H, Figure 3J, Figure 4C, Supplementary Figure 5, Supplementary Figure 6

Field-specific reporting

Please select the one below that is the best fit for your research. If you are not sure, read the appropriate sections before making your selection.

☒ Life sciences ☐ Behavioural & social sciences ☐ Ecological, evolutionary & environmental sciences

For a reference copy of the document with all sections, see nature.com/documents/nr-reporting-summary-flat.pdf

Life sciences study design

All studies must disclose on these points even when the disclosure is negative.

Sample size	The diameter of the PECL microfibers and the fiber spacings were determined by using scanning electron microscope and the software Image Pro Plus.
Data exclusions	No data were excluded from the analysis.
Replication	All experiments were verified the reproducibility successfully.
Randomization	The samples were allocated randomly into experimental groups.
Blinding	The investigators were blinded to group allocation during data collection and analysis.

Reporting for specific materials, systems and methods

We require information from authors about some types of materials, experimental systems and methods used in many studies. Here, indicate whether each material, system or method listed is relevant to your study. If you are not sure if a list item applies to your research, read the appropriate section before selecting a response.

Materials & experimental systems

n/a	Involved in the study	
☐	☒	Antibodies
☒	☐	Eukaryotic cell lines
☒	☐	Palaeontology
☐	☒	Animals and other organisms
☒	☐	Human research participants
☒	☐	Clinical data

Methods

n/a	Involved in the study	
☒	☐	ChIP-seq
☒	☐	Flow cytometry
☒	☐	MRI-based neuroimaging

Antibodies

Antibodies used	Vimentin: Proteintech; 10366-1-AP; Rabbit Polyclonal;<br>ALDH3A1:Proteintech; 15578-1-AP; Rabbit Polyclonal<br>Collagen VI: Abcam; ab182744; Rabbit Monoclonal;<br>b-actin: Proteintech; 20536-1-AP; Rabbit Polyclonal;<br>Goat Anti-Rabbit IgG H&L (Alexa Fluor® 488): Abcam; ab150077<br>HRP coupled goat anti-rabbit IgG(H+L): Proteintech; SA00001-2;
Validation	Vimentin: human, mouse, rat; WB, IHC, IF, FC, ELISA<br>ALDH3A1: human, mouse, rat; WB, IP, IHC, IF, FC, ELISA<br>Collagen VI: human, mouse, rat; WB, ICC/IF, IHC-P<br>b-actin: human, mouse, rat, zebrafish, monkey, goat, Hamster, pig, Plateau pika, swine; WB, IHC, IF, ELISA<br>Goat Anti-Rabbit IgG H&L (Alexa Fluor® 488): Rabbit IgG; ICC/IF, Flow Cyt, IHC-P, ELISA, IHC-Fr<br>HRP coupled goat anti-rabbit IgG(H+L): Rabbit IgG; ELISA,WB

Animals and other organisms

Policy information about studies involving animals; ARRIVE guidelines recommended for reporting animal research

Laboratory animals	SD rat, male, 4-week old
Wild animals	The study did not involve wild animals.
Field-collected samples	The study did not involve samples collected from the field.
Ethics oversight	The animals were maintained in a temperature-controlled environment (20 ± 1oC) with free access to food and water. All procedures were performed with the approval of the Animal Ethics Committee of Tsinghua University.

Note that full information on the approval of the study protocol must also be provided in the manuscript.
